# A Multilayered Magnetoelectric Transmitter with Suppressed Nonlinearity for Portable VLF Communication

**DOI:** 10.34133/research.0208

**Published:** 2023-09-15

**Authors:** Zhaoqiang Chu, Zhineng Mao, Kaixin Song, Shizhan Jiang, Shugang Min, Wei Dan, Chenyuan Yu, Meiyu Wu, Yinghui Ren, Zhichao Lu, Jie Jiao, Tianxiang Nan, Shuxiang Dong

**Affiliations:** ^1^ Qingdao Innovation and Development Base, Harbin Engineering University, Qingdao 266071, China.; ^2^ College of Underwater Acoustics Engineering, Harbin Engineering University, Harbin 150001, China.; ^3^ Songshan Lake Materials Laboratory, Dongguan, Guangdong 523808 China.; ^4^ Shanghai Institute of Ceramics, Chinese Academy of Sciences, Shanghai 201800, China.; ^5^ School of Integrated Circuits and Beijing National Research Center for Information Science and Technology (BNRist), Tsinghua University, Beijing 100084, China.; ^6^ College of Engineering, Peking University, Beijing 100871, China.

## Abstract

Acoustically actuated magnetoelectric (ME) antenna based on the efficient oscillation of magnetic dipoles has recently been considered as a promising solution for portable very-low-frequency communications. However, the severe nonlinear dynamic behavior in the case of strong-field excitation results in insufficient radiation capability and poor communication performance for a conventional ME antenna. In this work, we propose to suppress the nonlinearity of an ME antenna by neutralizing the spring-hardening effect in amorphous Metglas and the spring-softening effect in piezoelectric ceramics through an ME multilayered transmitter (ME-MLTx) design. With a driving voltage of 50 V_pp _ at the resonance frequency of 21.2 kHz, a magnetic flux density as high as 108 fT at a distance of 100 m is produced from a single ME-MLTx. In addition, ME-MLTx performs a decreased mechanical quality factor (*Q*_m_) less than 40.65, and, thus, a broadened bandwidth of 500 Hz is generated. Finally, a communication link transmitting binary American Standard Code for Information Interchange-coded message is built, which allows for an error-free communication with a distance of 18 m and a data rate of 300 bit/s in the presence of heavy environment noise. The communication distance can be further estimated over 100 m when using a femtotesla-class-inductive magnetic field receiver. The obtained results are believed to bring ME antennas one step closer to being applicable in very-low-frequency communications.

## Introduction

Modern antenna designs generally focus on carrier frequencies from megahertz to gigahertz for supporting high-data-rate wireless communication while keeping a portable antenna size. Stable low-frequency magnetic/electric field communication, which is immune from the high propagation loss, the multipath propagation, and the complexity of the underwater operating environment, i.e., low visibility and strong marine noise, enables the information interaction between miniaturized bionic robots and supports the wireless communication across the seawater [1–4]. However, a low-frequency (<30 kHz ) transmitter, which is both efficient and portable, cannot be constructed by traditionally accelerating electrons within a conductor [2]. In contrast, strain-powered mechanical antenna has recently been proposed and demonstrated to realize potable very-low-frequency (VLF) and ultralow-frequency communications. Various approaches have been examined to efficiently oscillate the diploes in dielectric or magnetic materials for radiating low-frequency electromagnetic field [5–10]. For example, electromagnetic motors are first considered to rotate a permanent magnet [11,12]. However, speed modulation in this case is suffered from the strong inertia, and extremely low data rate is usually resulted. Manipulating microscopic dipoles by piezoelectric driving can circumvent the large inertia of permanent magnets and can, thus, allow for high modulation rate. For example, Nan et al. [13] fabricated a miniaturized magnetoelectric (ME) antenna with a diameter of only 200 μm or λ_0_/593, which is 1 to 2 orders of magnitude smaller than electrically small antennas with their sizes typically over λ_0_/10. The bulk acoustic waves in ME heterostructures FeGaB/AlN produce magnetization oscillations of the ferromagnetic thin film, which then results in the radiation of electromagnetic waves at gigahertz.

Starting from those milestone works, numerous efforts are made to further increase the radiation capability and to finally improve the communication performance for mechanical ME antennas. Xu et al. [14] and Dong et al. [15] separately constructed a VLF transmitter with widely used 2-1 ME composites. Note that 2-1 ME composites with high direct ME coupling coefficient (*α*_ME_ > 2, 000 V/cm ∗ Oe) and low magentic noise (5.1 pT/rtHz) were explored first for magnetic sensing applications [16–19]. In [14,15], a 1 fT flux density can be produced theoretically at 200 and 260 m, respectively. However, strong nonlinear dynamic characteristics were clearly observed for such an ME resonator under high-field excitation and physical failure usually occurred after continuous operation [15,20–22]. More recently, Xiao et al. [23] reported a dual-driven ME antenna with enhanced transmission efficiency and broadened bandwidth. The authors performed a communication test with a distance of 2 m and a data rate of 120 bps. Its quality factor remains as high as 332, which implies that high nonlinearity would also appear in such a dual-driven ME antenna if the nonlinear spring constant is not zero.

The current researches focus on the possibility to build a wireless communication system with ME mechanical antennas. Communication distance and data rate are 2 cross-related parameters that should be considered equally. Through reading related literatures, it is found that a lock-in amplifier or a dynamic signal analyzer is normally used to detect the weak magnetic field from an ME transmitter and the working distance is usually limited to 2 to 3 m [15,23]. In those experiments, real baseband signal was not examined; thus, the antenna performance such as eye patters could not be assessed, and true information cannot be transmitted either. On the other hand, nonlinearity in ME antenna is usually ignored, and the continuous working performance has gained little attention [24–26]. Considering an antenna system, one must keep in mind that high field excitation will result in rapid heating generation and unexpected saturation [26]. Conventional ME devices such as magnetic sensors and magnetic memories typically operate in quasi-static and low-field excitation [27–29]. In these cases, ME devices are basically linear, and the above heating generation and saturation problems are not existed. However, in our previous studies, dynamic nonlinearity in 2-1-typed and 1-1-typed ME resonator, i.e., skew-distorted spectrum, jumping response, and hysteresis, was clearly observed because of the strong delta-E effect in piezomagnetic material Metglas, which greatly limited the power handling of an ME antenna and might deteriorate the communication performance [24,25]. Hence, new ME antenna architecture by considering the dynamic nonlinearity controlling and real transmitting process exploring should be built.

In this work, we propose an ME multilayered transmitter (ME-MLTx) with suppressed nonlinearity and enhanced communication performance. The ME-MLTx consists of 3 layers of PZT-5H ceramics and 2 layers of Metglas laminates, which are serving as the driving and loading phases, respectively. Piezoelectric layers are alternately glued together with magnetostrictive layers and excited in parallel to increase the inverse ME coupling coefficient. In addition, the strong spring-hardening behavior resulted from the delta-E effect in Metglas is effectively neutralized in an ME-MLTx by enlarging the dynamics contribution from piezoelectric phases. The ME-MLTx also performs a low mechanical quality factor of 40.65, which equally contributes to the suppression of the nonlinearity and effectively broadens the −3 dB bandwidth to be 500 Hz. Benefitting from the suppressed nonlinearity and the enlarged volume of the ferromagnetic layers, magnetic flux density as high as 108 fT at a distance of 100 m is produced from a single ME-MLTx with its excitation voltage limited to the linear range. Besides, a communication link transmitting binary American Standard Code for Information Interchange-coded message is successfully built, which allows for an error-free communication distance of 18 m and a data rate of 300 bps in the presence of heavy environment noise.

## Results

### Design and characterization of the ME-MLTx

**Fig. 1.**
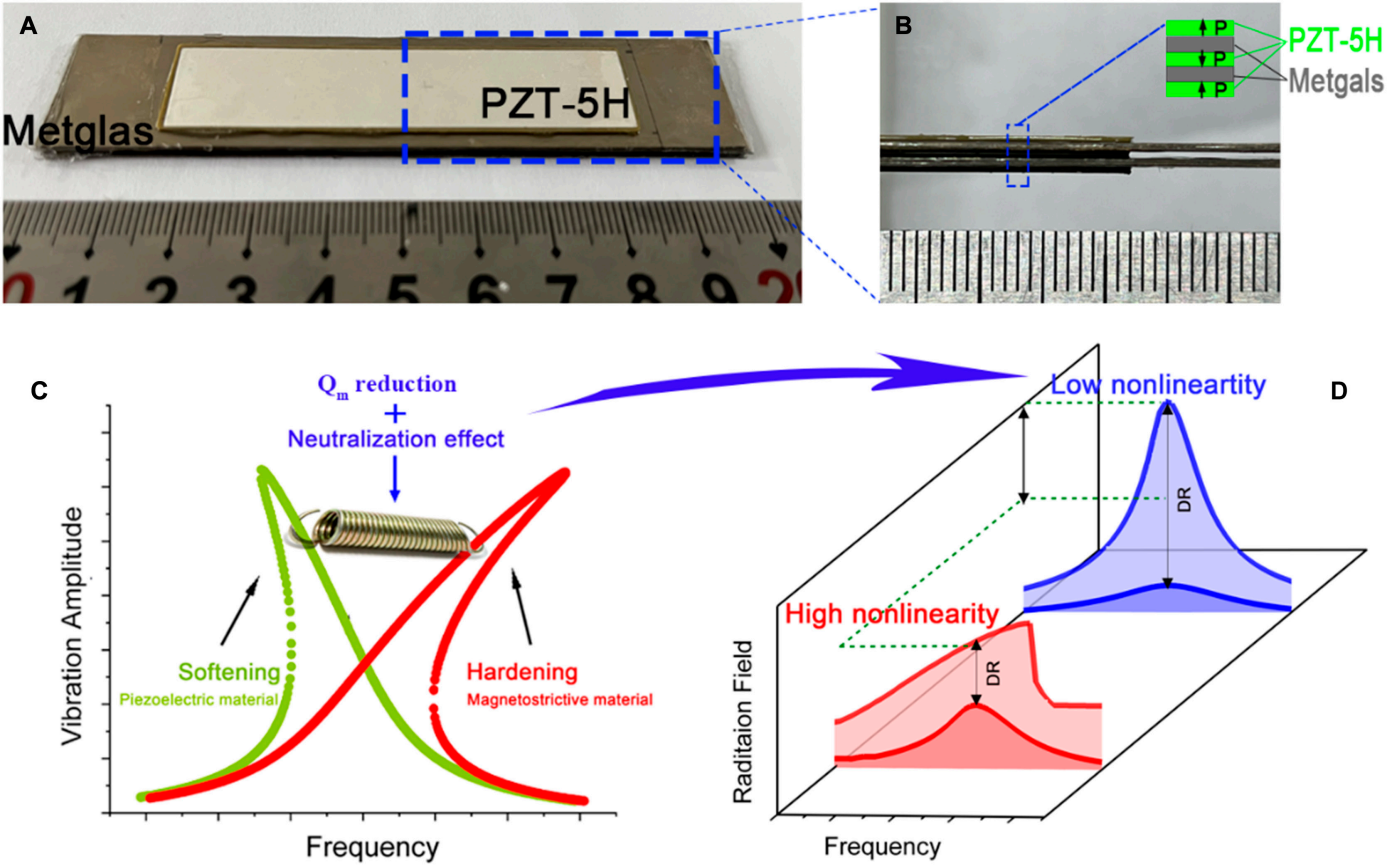
Sample photo and its behind design philosophy of the ME-MLTx. (A and B) The sample photo of an ME-MLTx (A) and its enlarged view of the longitudinal section (B). The inset provides a clear view of the polarization configuration of each piezoelectric element. (C) The schematic demonstration of our proposed neutralization effect between the spring-hardening effect in piezomagnetic material and the spring-softening effect in piezoelectric material. (D) The schematic demonstration of the improvement of dynamic range (DR) and the radiation capability by suppressing the nonlinearity in an ME antenna.

Conventional ME composites with enhanced direct ME coupling coefficient normally have a piezoelectric core sandwiched between 2 magnetostrictive laminates [30]. However, direct and converse ME effects are not fully reciprocal for a given ME resonator [31]. To be specific, direct ME coupling coefficient maximizes at the antiresonance frequency, while converse ME coupling gets the peak response at the resonance frequency for a same resonator. Increasing the volume ratio of magnetostrictive phases and enhancing the mechanical quality factor are usually utilized to strengthen direct ME coupling capability for a sandwich-structured ME resonator [32,33]. However, the corresponding converse ME coefficient is often poor when simply increasing the volume of loading phase, and strong dynamic nonlinearity will be easily resulted for a high *Q*_m_ resonator [15]. In a bid to obtain both strong converse ME coupling capability and enlarged linear power handling, we proposed an ME-MLTx as shown in Fig. 1A. The dimension of our used PZT-5H is 70 mm^length^ × 15 mm^width^ × 0.5 mm^thickness^, and the Metglas foil is tailored to 100 mm^length^ × 20 mm^width^ × 0.025 mm^thickness^. Figure 1B gives the longitudinal section of the ME-MLTx and shows the stack order of each functional layer. Here, 15 layers of Metglas foil are glued together to form a magnetostrictive laminate, and its total thickness is around 0.45 mm. The inset of Fig. 1B also marks the polarization configuration of each piezoelectric element. When looking into the dynamic characteristics of a resonator as we earlier reported in [24], a magnetostrictive resonator under an optimized magnetic bias tends to be a spring-hardening system since the Young’s moduli of Metglas will increase as we enlarge the excitation field [24], as shown in Fig. S1. On the contrary, a single piezoelectric resonator performs the spring-softening behavior in the case of strong-filed excitation [26,34]. The multilayered structure in the ME-MLTx can then bring a neutralization effect between the spring-hardening effect in piezomagnetic materials and the spring-softening effect in piezoelectric materials, and, thus, the suppressed nonlinearity is enabled (Fig. 1C). Figure 1D further schematically presents the improvement of the linear dynamic range and the radiation capability of an ME-MLTx by suppressing the dynamic nonlinearity.

**Fig. 2.**
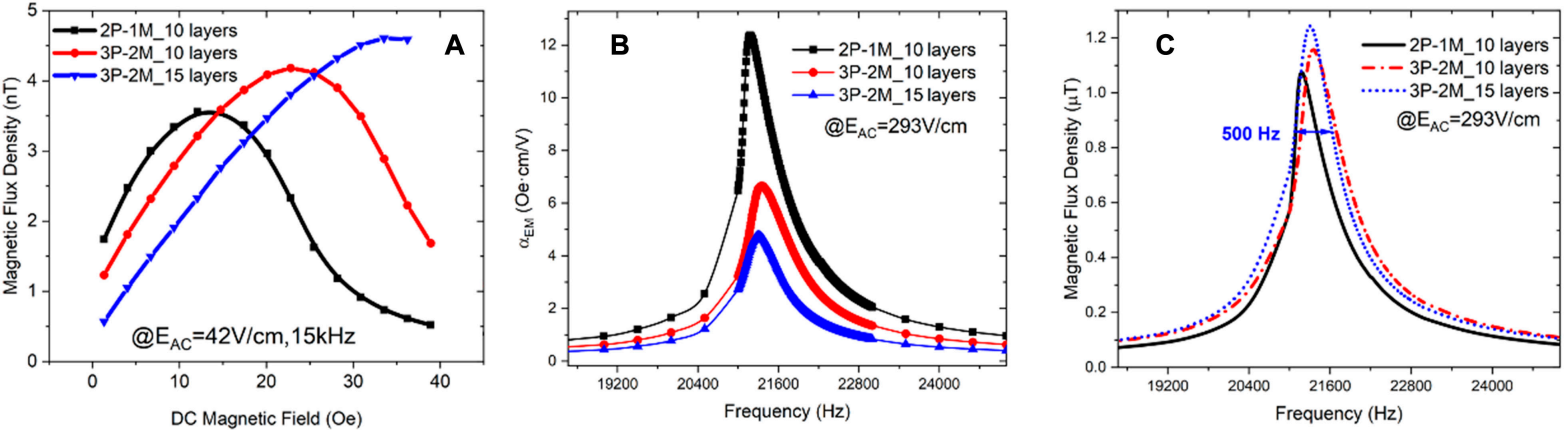
Characterization of the magnetic bias curve, the converse ME coupling coefficient, and the radiated magnetic flux density of the ME-MLTx. (A) Received magnetic flux density in air for 3 kinds of ME transmitter as a function of the applied bias magnetic field. (B and C) Comparison of the converse ME coupling coefficient (B) and the radiated magnetic flux density (C) for our fabricated transmitters. Here, the driven field is 293 V_pp_/cm.

Compared with a traditional 2-1-typed ME resonator, an improved stress transfer between piezoelectric phases and piezomagnetic phases for an ME-MLTx was verified by finite element analysis as given in Fig. S2. To further demonstrate the nonlinearity manipulation for an ME-MLTx, we then compared 3 kinds of ME transmitters. The first kind has a sandwiched structure with the magnetostrictive core embedded in piezoelectric phases. The magnetostrictive core consists of 10 Metglas foils, and this kind of ME transmitter is labeled as 2P-1M_10. Here, the first number represents the number of used piezoelectric layers, while the second and the third number stands for the number of used piezomagnetic laminates and the number of Metglas foil in each piezomagnetic laminate. The other 2 kinds of ME-MLTx are correspondingly tagged as 3P-2M_10 and 3P-2M_15, respectively. Figure S3 then modeled the magnetic flux density distribution in the longitudinal section with a constant background field of 4 mT. It is found that strong magnetic flux concentration effect appears in magnetostrictive laminate for transmitter 2P-1M_10. However, ME-MLTx performs much weakened flux concentration effect, especially for transmitter 3P-2M_15. We then measured the generated magnetic flux density at a distance of 30 cm away as a function of applied dc magnetic bias field when exciting the transmitters under off-resonance frequency (see Fig. 2A). The optimized bias field increases to 25 and 32 Oe for transmitter 3P-2M_10 and 3P-2M_15, respectively, while conventional transmitter 2P-1M_10 reaches the peak response under optimized bias field as low as 12 Oe. The increase in magnetic bias field in ME-MLTx is resulted from the weakened magnetic flux concentration effect as illustrated in Fig. S3A to C. Then, ME-MLTx was equipped with a pair of permanent magnets to provide the required bias field. Figure 2B compares the converse ME coupling coefficient as a function of the driving frequency. Transmitter 2P-1M_10 can achieve a coupling coefficient as high as 12.5 Oe·cm/V. In contrast, the ME-MLTx 3P-2M_15 has a lower coefficient of approximately 4.8 Oe*cm/V, primarily due to the increased loading. However, transmitter 3P-2M_15 generates a higher flux density, as illustrated in Fig. 2C. This is because the radiated flux density under a certain excitation field is the product of the effective volume of the magnetostrictive layers and the converse coupling coefficient at resonance frequencies. In addition, note that the converse ME coupling coefficient of transmitter 3P-2M_15 is also enhanced compared with that in previously reported 2-1-typed ME transmitter [15].

**Fig. 3.**
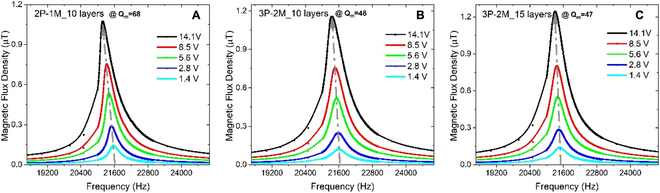
Suppressed nonlinearity in ME-MLTx. (A to C) The frequency–response curve of the received magnetic flux density for transmitter 2P-1M_10 (A), 3P-2M_10 (B), and 3P-2M_15 (C). Here, the excitation voltage is generated by a lock-in amplifier in combination with a linear power amplifier. The induced voltage from the coil receiver is directly detected by the same lock-in amplifier. The mechanical quality factor was calculated through resonance frequency divided by the −3 dB bandwidth under 2.8 V_pp_ excitation. A LabVIEW program is used to implement the sweeping experiment.

Figure 3 then characterized the frequency–response curve of the received magnetic flux density. Strong spring-hardening phenomenon was previously observed for a high-*Q*_m_ ME resonator with its resonance frequency obviously shifting to a higher value because of the delta-E effect of piezomagnetic material Metglas [24,25]. It should be noted here that piezoelectric phase prevails in transmitter 2P-1M_10. Because of the spring-softening behavior in piezoelectric phases, the resonance frequency of transmitter 2P-1M_10 shows a decrease trend as the driving voltage increases as shown in Fig. 3A [24,26]. By comparison, a modest frequency shifting occurs in transmitter 3P-2M_10, and the proposed transmitter 3P-2M_15 even performs a linear response, which further manifests that ME-MLTx has a lowered nonlinearity and, thus, the linear power handing is effectively enlarged. Note that previous work normally excited the transmitter with an electric field higher than the linear threshold at a fixed frequency point and strong nonlinearity was actually resulted in this case. The impedance and phase spectra for those 3 kinds of ME transmitter were also compared in Fig. S4. Figure S5 further revealed the evolution of the nonlinear behavior as we gradually increased the volume ratio of piezomagnetic phase, and the neutralization effect between the spring-hardening effect in piezomagnetic materials and the spring-softening effect in piezoelectric magnetostrictive materials was experimentally verified. As it can be seen from Fig. 3 and Fig. S4, much decreased mechanical quality factor is resulted in ME-MLTx because of the enlarged loading, which also contributes to the suppressed nonlinearity and enables a broadened −3 dB bandwidth.

### Magnetic radiation of the ME-MLTx

**Fig. 4.**
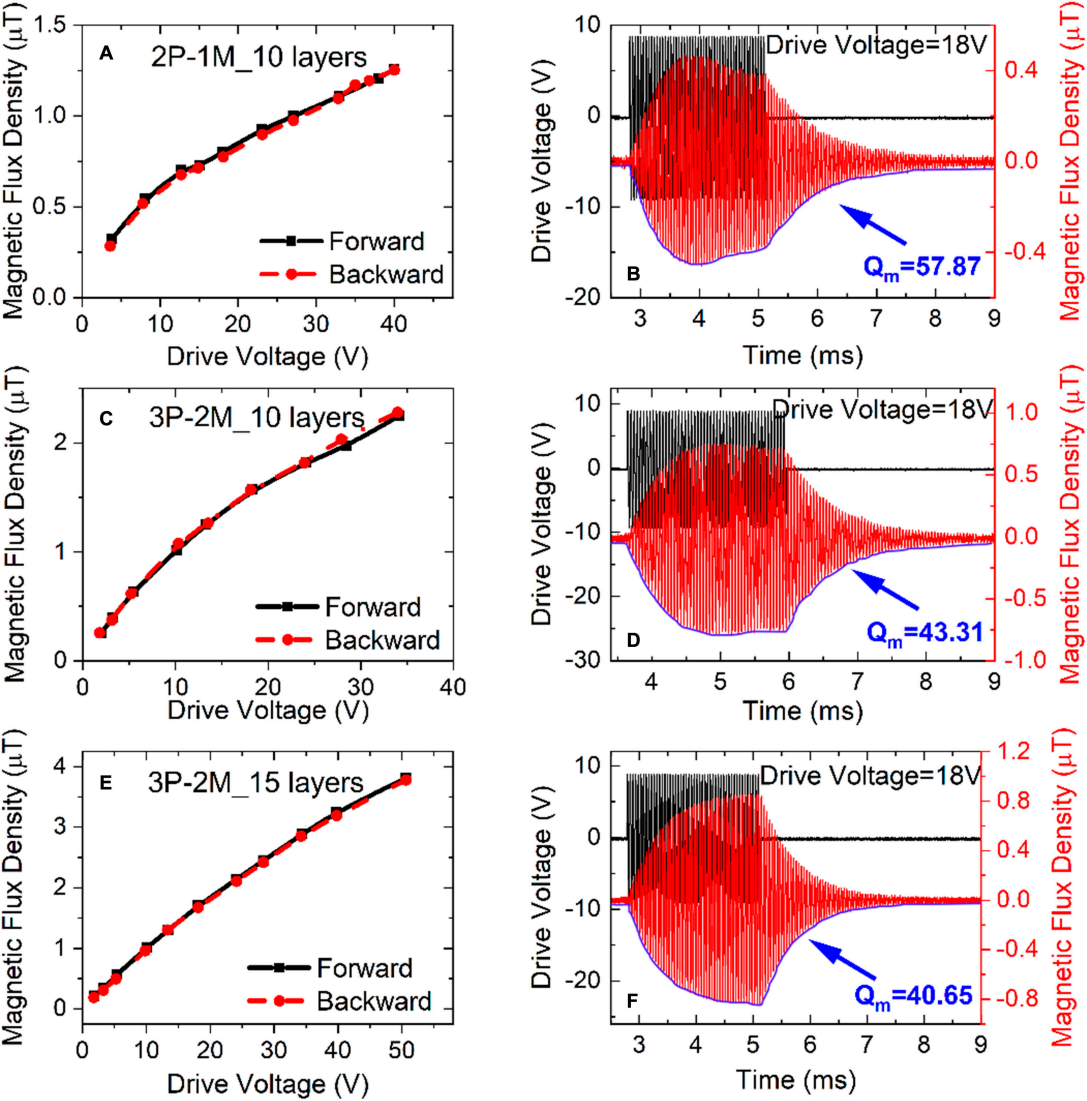
Generated magnetic flux density and vibration ring-down characteristic for the ME-MLTx as a function of the excited voltage. (A, C, and E) Received magnetic flux density at 30-cm distance as a function of the amplitude of the burst signal for transmitter 2P-1M_10 (A), 3P-2M_10 (C), and 3P-2M_15 (E). Here transmitters are excited with a weak burs field first to fix the resonance frequency. (B, D, and F) Voltage of the applied burst signal and the corresponding response waveform of the received magnetic flux density for transmitter 2P-1M_10 (B), 3P-2M_10 (D), and 3P-2M_15 (F). Here, the quality factor was calculated on the basis of free vibrations in the tail when 18 V_pp_ excitation signal is finished.

The bias–response curve and the ME coupling coefficient in Fig. 2A and B were measured with a weak driving voltage less than 15 V_pp_. To fully identify the radiation capability, it is necessary to characterize the tolerable electric field intensity for a linear ME transmitter. Figure S6 shows the graphical setup for measuring the radiation capability of an ME transmitter. The received magnetic flux density as a function of the driving voltage is then given in Fig. 4. For conventional ME transmitter 2P-1M_10, the received magnetic flux density is apparently growing nonlinearly within a driving voltage of 40 V_pp_, and the envelope curve of the ring-down waveform displays aberration even with a weak driving voltage of 18 V_pp_ (as shown in Fig. 4A and B). In contrast, the ME-MLTx performs enhanced radiation performance (see Fig. 4C to F). For transmitter 3P-2M_15 in particular, the received magnetic flux density is almost increasing linearly with the driving voltage limited to 40 V_pp_, and a normal ring-down waveform is observed as depicted in Fig. 3E and F. Considering a freely decaying oscillation, the mechanical quality factor of 40.65 is extracted from the ring-down waveform in Fig. 4F, while transmitter 2P-1M_10 has a much higher-quality factor as shown in Fig. 4B, which agrees well with the calculated results in Fig. S4. Here, we demonstrate that ME-MLTx is able to keep a linear resonance under 40 to 50 V_pp_ driving voltage.

**Fig. 5.**
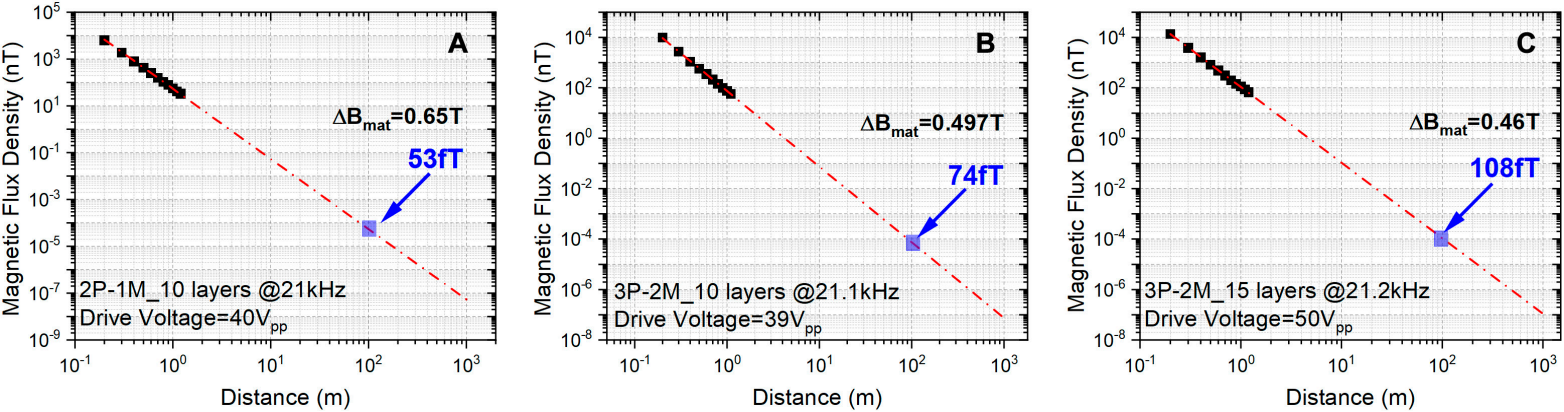
Generated magnetic flux density for the ME-MLTx at different distances. Measured *B* field versus range for transmitter 2P-1M_10 (A), 3P-2M_10 (B), and 3P-2M_15 (C). Here, the modulation intensity ∆*B*_mat_ of the oscillated magnetization in piezomagnetic layers was calculated to be 0.65, 0.497, and 0.46 T, respectively. In (C), the transmitter 3P-2M_15 was excited with a voltage of 50 V_pp_, and the linear fitting curve was obtained by considering a theoretical attenuation of the radiated magnetic field as 1/*r* ^3^ in the near-field zone for a magnetic dipole. The measured data also agreed well with the fitted results.

In a bid to ultimately evaluate the radiation capability of our proposed ME-MLTx, we measured the received magnetic flux density at different distances and extracted the modulation intensity ∆*B*_mat_ of the oscillated magnetization in piezomagnetic layers, as compared in Fig. 5A to C. According to the results in Fig. 5C, transmitter 3P-2M_15 was excited with 50 V_pp_ voltage in resonance. The measured flux density at 1 m distance in free space reaches 112 nT, and a predicted flux density of 108 fT at 100 m distance is allowed, which is almost 5 times the reported magnitude in [8,15]. In addition, the ∆*B*_mat_ of ME-MLTx 3P-2M_15 is calculated to be 0.46 T (see Materials and Methods section for detailed definition and calculation for modulation intensity ∆*B*_mat_), which indicates a huge potential for further enhanced radiation capability since the saturated magnetization of Metglas (FeCoSiB) reaches as high as 1.74T.

### Continuous working performance of the ME-MLTx

**Fig. 6.**
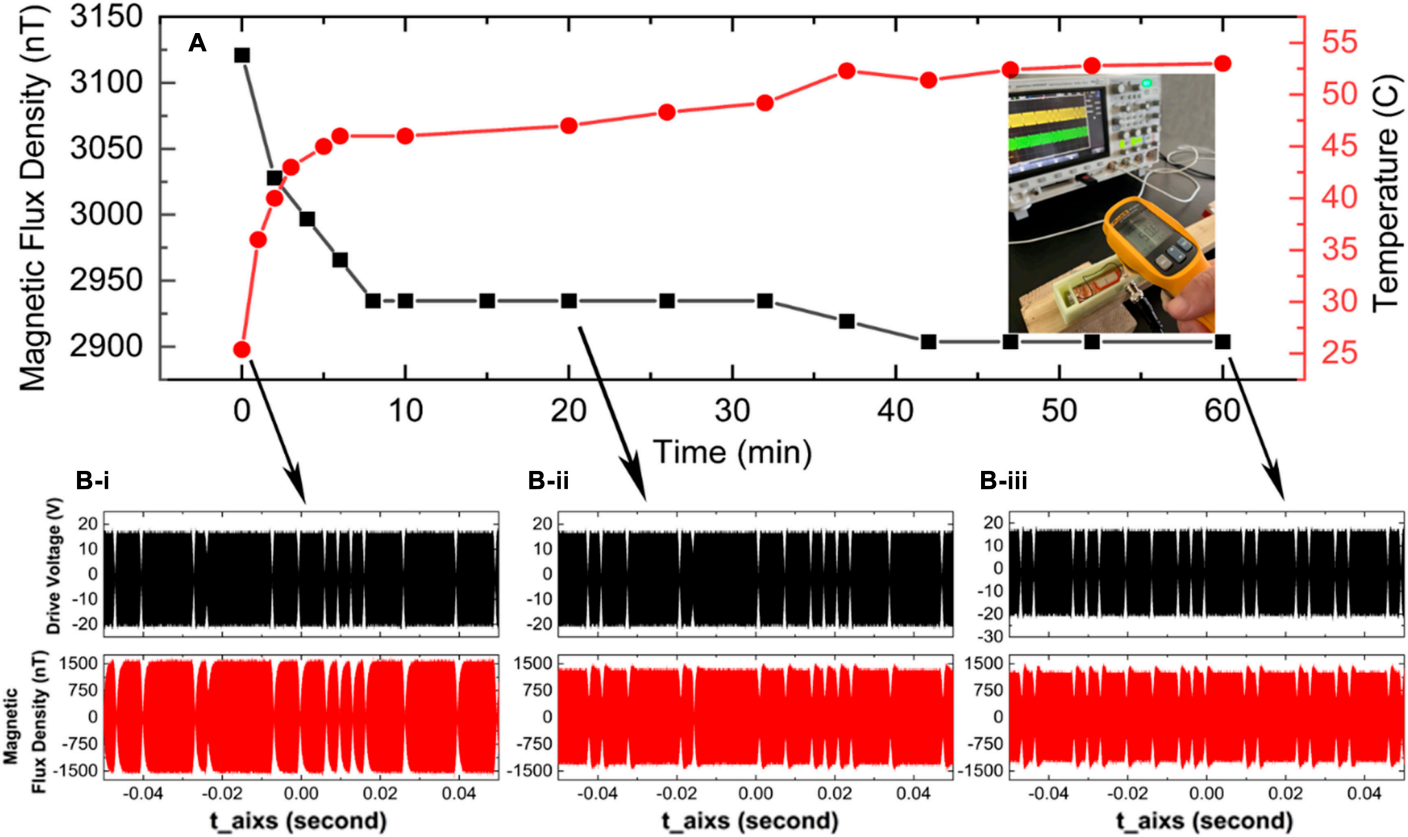
Continuous working performance of the ME-MLTx 3P-2M_15. (A) The measured flux density at 30 cm away and the surface temperature for ME-MLTx 3P-2M_15 during the continuous working. (B) The time-domain waveform of the sending BPSK sequence and the received magnetic signal during the course of the continuous work.

The continuous working performance of ME-MLTx 3P-2M_15 was then tested as shown in Fig. 6A. Here, the ME antenna kept working for 1 h. A quick temperature rise was observed within the first 10 min, and the produced magnetic flux density at 30 cm away correspondingly decreased from 3,125 to 2,940 nT. During the next 50 min, the temperature at the node position gradually stabilized at 53 °C, and the radiation performance showed a very minor deterioration. Figure 6B recorded the time-domain waveform of the sending binary phase-shift keying (BPSK) sequence and the received magnetic signal. Here, an important observation is the balance between the rates of warming and cooling for the ME-MLTx 3P-2M_15 under 40 V_pp_ excitation, and, thus, the ultimate temperature can stabilize at 53 °C. Higher temperature could be expected if we further increase the excitation voltage. As the Curie temperature for piezoelectric material PZT-5H is normally higher than 100 °C, we can safely conclude that the ME-MLTx is able to keep continuous work and the performance can be even fully recovered once the antenna stops working to decrease the temperature. In general, the quick temperature rise could also lead to a spring softening behavior, and, thus, the resonance frequency will be changed during the working. On this basis, keeping operating within the linear range and decreasing the loss for an ME antenna are critical, and an acceptable temperature rise should be considered for practical communication application.

### VLF communication based on the ME-MLTx

**Fig. 7.**
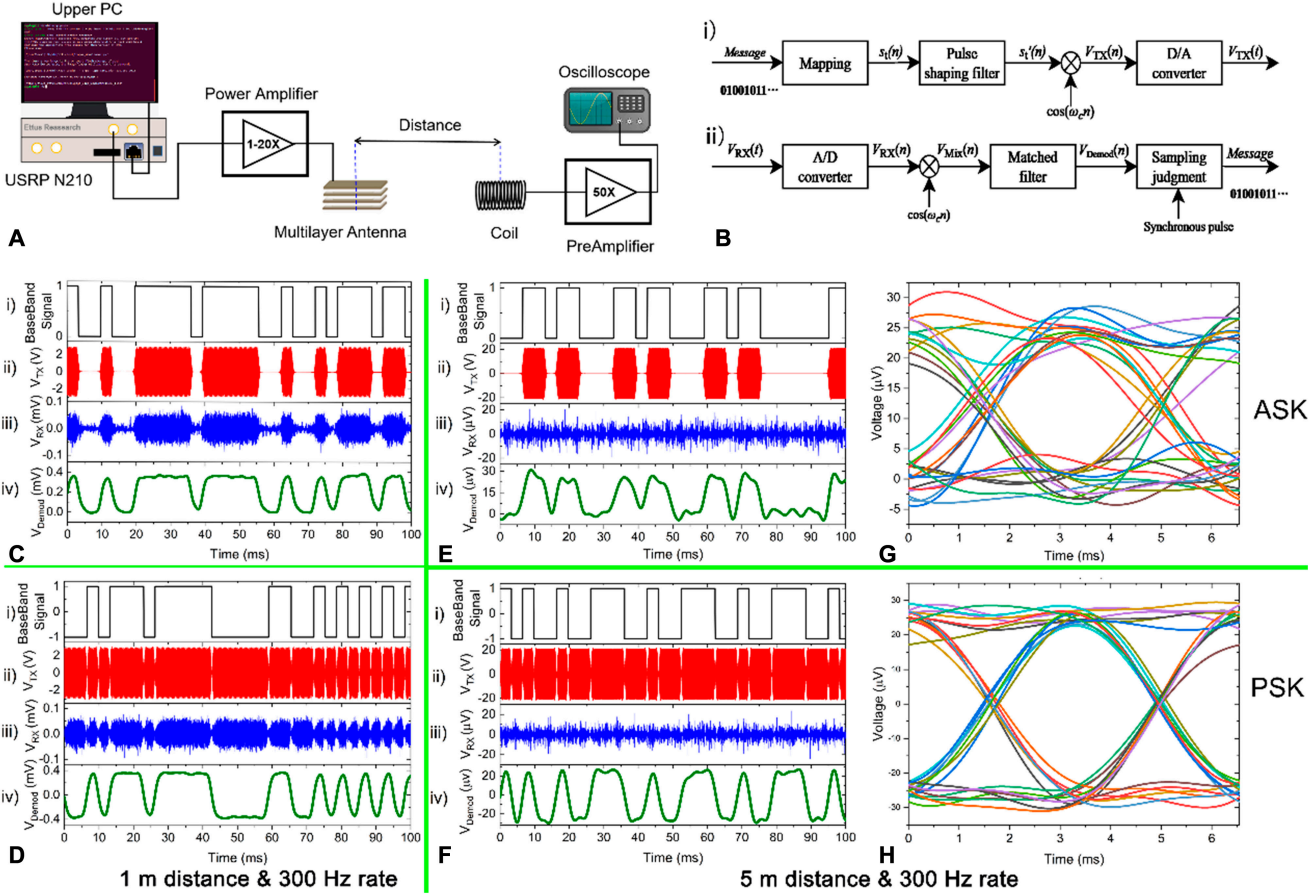
System configuration, modulation schemes, and communication results of our proposed ME-MLTx. (A) System configuration for transmitting and receiving binary message by VLF magnetic communication. Here, the used binary message consists of 300 bits, and it is repeatedly sent out by the ME-MLTx. A small air-core coil receiver was used to capture the radiated magnetic field. (B) The block diagram for generation and coherent detection of the modulated signal. Here, both BASK and BPSK modulation schemes are tested and compared. (C and D) VLF communication implementation at 1-m distance by ASK modulation (C) and PSK modulation (D). From top to bottom: (i) binary baseband signal *s*_t_(*n*) with a symbol rate of 300 bps, (ii) the modulated transmitting signal *V*_TX_(*n*), (iii) the received signal from the coil receiver *V*_RX_(*n*), and (iv) the demodulated voltage signal *V*_Demod_(*n*). Note that matching filter dramatically enhanced the SNR and, thus, the voltage level of *V*_Demod_(*n*) was much higher than that of *V*_RX_(*n*) given a same noise level. (E and F) VLF communication verification at 5-m distance for ASK modulation (E) and PSK modulation (F). (G and H) The corresponding eye diagram for detected signal at 5-m distance by BASK modulation (G) and BPSK modulation (H).

Figure S7 gives the near-field radiation patterns in plane for ME-MLTx 3P-2M_15 under 20 V_pp_ excitation at resonance frequency. A perfect splay pattern is observed, and a large-angle coverage can be allowed. Then, a VLF communication link based on our proposed ME-MLTx and a coil receiver is built to transmitting and receiving a binary message. Here, the coil receiver is oriented toward 0°or 180° direction (see Fig. S7). Figure 7A shows the system implementation configuration. Signal modulation is finished by a Universal Software Radio Peripheral N210 platform that is equipped with a low-frequency transmit daughterboard and can operate from 0 to 30 MHz. Then, the modulated signal is amplified by a linear power amplifier before it is connected to the ME-MLTx. A home-made air-core coil with the dimension of ø34 mm × 60 mm^length^ is used to detect the radiated magnetic field. At the receiving side, the induced voltage from the coil is first passed through the voltage preamplifier (SR560, Stanford Research, USA). Finally, data postprocessing is proceeded to finish the demodulation based on the recorded voltage signal from an oscilloscope.

Figure 7B gives the block diagram for generation and coherent detection of the modulated signal. In [20], direct antenna modulation (DAM) technique was proposed to decouple the bandwidth from the high mechanical quality factor. A 7 Hz modulation depth was realized, and frequency shift keying (FSK) rate up to ~200 Hz was enabled for a high-*Q* piezoelectric mechanical antenna, which has a narrow −3 dB bandwidth of 84 mHz. In [9], binary FSK modulation without DAM assistance is conducted within the −3 dB bandwidth of the PZT resonator. Because of the high-quality factor and, thus, the narrow −3 dB bandwidth of 30 Hz, low FSK rate was resulted. Here, benefiting from high −3 dB bandwidth of 500 Hz in the ME-MLTx, both BASK (binary amplitude-shift keying) and BPSK modulation schemes can be used directly to achieve higher bandwidth efficiency and to remove additional assistance in DAM technique. The block diagram for generation and coherent detection of the modulated signal are given in Figs. 6B-i and 7B-i, respectively. Since ME antenna is typically a band-limited system, severe signal distortion and high bit error rate would result when sending a burst sequence. Here, a shaping filter is utilized to concentrate the signal energy within a narrow bandwidth, and a matched filter is performed on the digital mixing signal *V*_Mix_(*n*) before the sampling judgement to maximize the signal-to-noise ratio (SNR) and to reduce the intersymbol interference. The overall modulation and demodulation scheme are illustrated in Fig. S8.

We first examined the VLF communication performance when fixing the communication distance and the symbol rate to be 1 m and 300 bps, respectively. The corresponding baseband signal, the modulated transmitting signal, the induced voltage signal, and the demodulated signal are separately provided from top to bottom in Fig. 7C and D for BASK and BPSK modulation, respectively. Here, the driving voltage is only 5 V_pp_, and the binary bit stream can be successfully demodulated even without data postprocessing. Then, we enlarged the communication distance to be 5 m, and experimental results are given in Fig. 7E to H. Here, the driving voltage increases to 40 V_pp_, and a linear response can be still kept as demonstrated in Fig. 4E. Because of the high environment noise under laboratory condition, it is hard to identify the symbol stream directly from the induced voltage from the coil receiver (see Fig. 7E-iii and F-iii). However, the symbol sequence is clearly decoded when we apply the demodulation scheme as shown in Fig. 7B-ii. As can be seen from the demodulated signal, BPSK scheme is found to have enhanced SNR, which can be attributed to its higher bandwidth efficiency compared with BASK scheme. The system performance is further demonstrated through the obtained eye diagram for both BASK (Fig. 7G) and BPSK (Fig. 7H) modulation. It is clear the time variation of zero crossing, and the SNR are greatly improved in the case of BPSK modulation; it is believed longer communication distance could be allowed with the same communication system. Figure S9 then provides the test results at a communication distance of 7 m and a remained symbol rate of 300 bps. Although decreased SNR is resulted, the symbol sequence is still identified error-free in the presence of heavy environment noise. In addition, the symbol rate dependence of communication performance was also studied as depicted in Figs. S10 and S11.

**Fig. 8.**
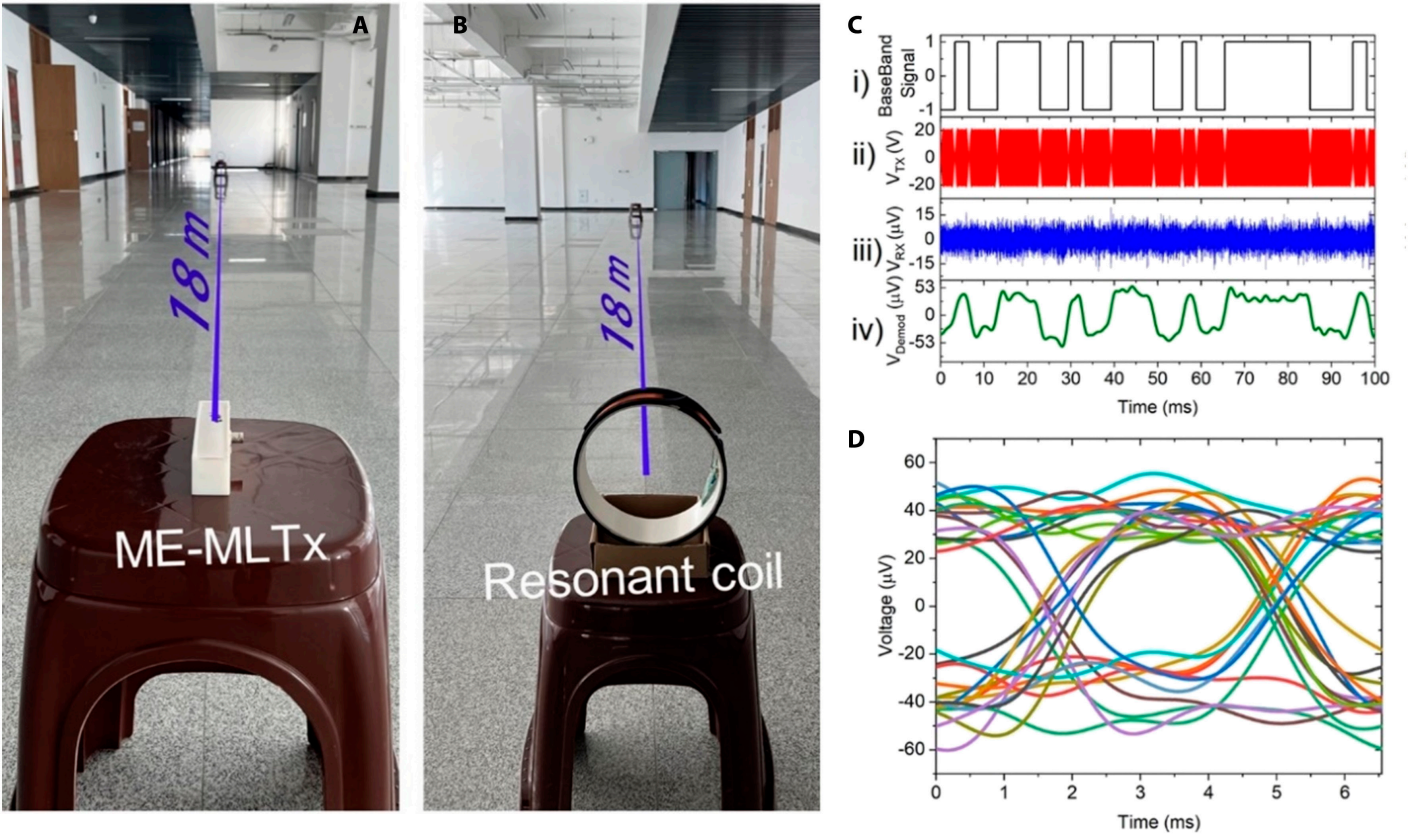
Long-distance communication performance verification in the presence of heavy laboratory noise of our proposed ME-MLTx. (A and B) The experimental setup in a laboratory building hallway. (C) The recorded transmitting sequence, driving and receiving voltage waveform, and the demodulated signal. (D) The obtained eye diagram for detected signal at 18-m distance by BPSK modulation.

In the field of communication field, product of the communication distance and the data rate is normally used to evaluate the system’s communication performance. Here, the product of 7 m × 300 bps is proved, and further increase in the communication distance is greatly limited by the receiver performance. To verify the long-distance communication performance, a tuning air-core coil receiver with a bigger diameter of 200 mm is used. The impedance curve for our used receiving coil is given in Fig. S12. The −3 dB bandwidth of the receiving coil can be modulated to cover the signal bandwidth by changing the resistance in parallel, and the current −3 dB bandwidth of 900 Hz for our used coil is wide enough to implement signal receiving. The symbol sequence can be directly read from the voltage waveform without any data processing in the case of 15 m communication distance (see Fig. S13 and Movie S1). When the distance further increased to 18 m (see Fig. 8A and B), the symbol sequence with 300 bps rate could be also identified error-free in the presence of environment noise with the demodulation scheme as shown in Fig. 7B-ii (see Fig. 8C and D). Such a communication performance in laboratory environment is first reported in the field of ME antenna to the best of our knowledge. It should be also noticed that random symbol sequence is also first examined here to verify the VLF communication application based on an ME antenna, while single-frequency signal or repeated burst signal was normally used to demonstrate the communication performance in previous reports [15,23]. Given the noise level of the environment and the detection ability of a receiver can be 20 to 30 fT/rtHz and 50 fT, respectively, our proposed ME-MLTx is believed to support communication distance as far as 100 m in the air.

## Discussion

In summary, we showed in this work how to improve the radiation capability and the communication performance for an ME antenna. An ME-MLTx was proposed and fabricated. The dynamic nonlinearity was well controlled by neutralizing the spring-hardening effect in amorphous Metglas and the spring-softening effect in piezoelectric ceramics. The ME-MLTx also performed a decreased mechanical quality factor less than 50 because of the increased load damping, which enabled a broadened bandwidth of 500 Hz and further suppressed the nonlinearity. As a result, we successfully excited the ME-MLTx linearly within 40 V_pp_ excitation voltage at the resonance frequency. In addition, the coupling coefficient in the ME-MLTx reached 4.8 Oe*cm/V, and the volume of piezomagnetic phase increased to 1.5 cm^3^. The combination of the above factors produced an enhanced radiation capability, and a flux density of 108 fT at 100 m distance could be expected for a single ME-MLTx. Meanwhile, we built a communication system based on the ME-MLTx. Random binary message with a symbol rate of 300 bps was transmitted and received error-free for the first time at 18 m communication distance via a resonant coil receiver in the presence of heavy laboratory noise. Moreover, we observed a quick temperature rise for the ME-MLTx during the continuous working, and, thus, an ME antenna with a weak spring-hardening effect might be favored to neutralize the spring-softening behavior caused by the heat generation for practical communication applications. It is believed that the concept of the design of the multilayered structure and the nonlinearity control method in this work will open up new dimensions in ME antenna’s study, and the preliminarily obtained communication performance indicates great potential for the engineering application of an ME antenna.

With respect to the optimized volume ratio of piezomagnetic phases, 3 factors should be considered including the nonlinearity suppressing, coupling efficiency enhancing, and the radiation intensity increasing. Since those 3 factors are interactive on each other, we first focused the nonlinearity suppressing in this work, and the optimized layer number of Metglas is 15 for transmitter ME-MLTx. In the coming research, we will further study the volume ratio optimization and the geometry design in the hope of reaching the highest radiation capability.

## Materials and Methods

### Fabrication and characterization of the ME-MLTx

The piezoelectric material selected in this paper is PZT-5H with the dimension of 70 mm^length^ × 15 mm^width^ × 0.48 mm^thickness^ and the piezomagnetic material is FeCoSiB (Vacuumscheltze GmbH & Co. KG, Germany) with tailored dimension of 100 mm^length^ × 20 mm^width^ × 0.025mm^thickness^. All the functional layers are glued together by epoxy resin (105/206, WEST SYSTEM, USA) at room temperature, and permanent magnets with a diameter of 20 mm were used to provide the optimized dc bias magnetic field. All the components above were packaged prior to properties measurement.

The converse ME coupling coefficient *α*_CME_ was measured at room temperature using a home-made setup. The distance between the coil receiver and the ME-MLTx is fixed as 30 cm. The excitation voltage *V*_exc_ (=2 V_rms_) was generated via a lock-in amplifier (SR860, Stanford Research, USA), and the induced voltage in the coil receiver *V*_coil_ was also detected with the same lock-in amplifier. To obtain the modulation intensity ∆*B*_mat_ of the oscillated magnetization, the radiated flux density at 30-cm distance *B*_air_ was first calculated by the following equation,

$$B_{\mathrm{air}}=\frac{V_{\text{coil}}}{2*\text{πfNS}}$$ (1)

where *N* and *S* represent the number of turns and the sectional area of the receiving coil. Then, the ∆*B*_mat_ defined as the oscillated magnetization intensity occurred in Metglas laminates could be derived from the theoretical attenuation of the radiated magnetic field as 1/*r* ^3^ in the near field zone.

$$B_{\mathrm{air}}=\frac{∆B_{\mathrm{mat}}*V_{\mathrm{mat}}}{2\pi r^{3}}\sqrt{1+r^{2}\beta^{2}},$$ (2)

where *V*_mat_ stands for the volume of piezomagnetic material and *β* is the free space wave number. Finally, the converse ME coupling coefficient *α*_CME_ can be defined as the ratio of the resulting magnetic flux density ∆*B*_mat_ to the applied electric field *V*_exc_/*d*. Here, *d* is the thickness of each PZT plate. The excitation frequency was swept through a LabVIEW program.

### Ring-down curve measurement and *Q*_m_ fitting

The period and the number of cycles for our used burst voltage in Fig. 4 are 500 ms and 50, respectively. Here, a voltage preamplifier was used for directly reading the induced voltage via an oscilloscope. During the experiment, the voltage gain was set as 50, and the passband was from 3 to 100 kHz. The mechanical quality factor *Q*_m_ was calculated on the basis of free vibrations in the tail when 18 V_pp_ excitation signal is finished, as shown in Fig. 4. The logarithmic decrement *Λ* was first obtained as follows

$$\Lambda =\text{log}\left(\frac{V_{t\left(n\right)}}{V_{t\left(n+1\right)}}\right),$$ (3)

where *V*_*t*(*n*)_ is the induced voltage at the *n*th peak in the ring-down curve. To improve the accuracy, we considered multiple periods. Then, we calculated the damping coefficient *ξ* and the quality factor *Q*_m_ based on the following relation

$$\Lambda =\frac{2\pi \xi }{\sqrt{1-\xi^{2}}},$$ (4)

$$Q_{m}=\frac{1}{2\xi },$$ (5)

### Signal modulation and demodulation scheme

In the modulation part, suppose the binary message to be sent is *b_i_* (*b_i_* = 0/1 for BASK and *b_i_* = −1/1 for BPSK), and then the baseband signal *s*_t_(*n*) can be expressed as follows

$$s_{t}\left(n\right)=\sum_{i}b_{i}\cdot {\mathrm{rect}}_{M}\left(n-\mathrm{iM}\right),$$ (6)

where

$${\mathrm{rect}}_{M}\left(n\right)=\left\{\begin{array}{cc} 1 & n=0,1,\ldots ,M-1\\ 0 & \text{others} \end{array}\right.,$$ (7)

and *M* is the number of samples in one pulse period. Then, the digital baseband signal *s_t_* (*n*) is filtered by a shaping filter *h*(*n*) to match the ring-down characteristic of the ME-MLTx. *h*(*n*) can be a square-root raised cosine finite impulse response filter with the roll-off factor and the span to be 0.9 and 8, respectively. To reduce intersymbol interference, the same square-root raised cosine finite impulse response filter is used at the receiver end. Thus, the digital modulation signal *V*_TX_(*n*) can be described as

$$V_{\mathrm{TX}}\left(n\right)=s_{t}^{\prime }\left(n\right)\cdot \text{cos}\left(\omega_{c}n\right),$$ (8)

where *ω*_c_ is the digital carrier frequency. Finally, the analog modulation signal *V*_TX_(*t*) output through a digital-to-analog converter is sent to the ME-MLTx after passing through the power amplifier.

In the demodulation part, the digital received signal *V*_RX_(*n*) is obtained by sampling the induced voltage signal from the coil receiver, which can be expressed as

$$V_{\mathrm{RX}}\left(n\right)=L\cdot s_{t}^{\prime }\left(n\right)\cdot \text{cos}\left(\omega_{c}n\right)+N\left(n\right),$$ (9)

where *L* represents the path attenuation and *N*(*n*) is the receiving system noise. Then, digital mixing signal *V*_Mix_(*n*) can be get by

$$\begin{array}{ll} V_{\mathrm{Mix}}\left(n\right) & =V_{\mathrm{RX}}\left(n\right)\cdot \cos\left(\omega_{c}n\right)\\ & =L\cdot s_{t}^{\prime }\left(n\right)\cdot \cos^{2}\left(\omega_{c}n\right)+N^{\prime }\left(n\right)\\ & =\frac{L}{2}\cdot s_{t}^{\prime }\left(n\right)+\frac{L}{2}\cdot s_{t}^{\prime }\left(n\right)\cdot \cos\left(2\omega_{c}n\right)+N^{\prime }\left(n\right) \end{array}$$ (10)

The mixed signal *V*_Mix_(*n*) is filtered by a matched filter to remove the frequency-doubling signal cos(2*ω*_c_ *n*) and to suppress the noise *N'*(*n*). Thus, the extracted digital demodulated signal *V*_Demod_(*n*) can be expressed as

$$V_{\text{Demod}}\left(n\right)=L^{\prime }\cdot s_{t}^{\prime }\left(n\right)+N^{\prime \prime }\left(n\right).$$ (11)

Finally, the binary message is obtained by judging the digital demodulated signal *V*_Demod_(*n*) at the maximum value of the symbol by the synchronous pulses.

## Data Availability

All data needed to evaluate the conclusions in the paper are present in the paper and/or the Supplementary Materials. Any additional datasets, analysis details, and material recipes may be requested from the authors.

## Supplementary Materials

Supplementary 1 Fig. S1. The calculated Young’s modulus of Metglas as a function of applied magnetic field and stress field. Fig. S2. The stress distribution and the resonance response for traditional 2-1-typed and our proposed multilayered ME resonator. Fig. S3. Magnetic flux density distribution for the ME-MLTx. Fig. S4. The impedance and phase spectra for transmitter 2P-1M_10, 3P-2M_10 and 3P-2M_15. Fig. S5. Nonlinearity evolution. Fig. S6. Characterization of the nonlinearity and the radiation capability of the ME-MLTx. Fig. S7. Near-field radiation pattern in horizontal plane for ME-MLTx 3P-2M_15. Fig. S8. The overall transmitting and receiving scheme for BPSK modulation. Fig. S9. VLF communication implementation at 7-m distance by BPSK modulation with a symbol rate of 300 bps. Fig. S10. Influence of the data rate on obtained eye diagrams for BASK modulation. Fig. S11. Influence of the data rate on obtained eye diagrams for BPSK modulation. Fig. S12. The impedance curve for our used tuning coil. Fig. S13. Verification of long-distance VLF communication. Movie S1. Fifteen-meter VLF communication experiment.
