# Supplementary material for: A Multilayered Magnetoelectric Transmitter with Suppressed Nonlinearity for Portable VLF Communication
Source: Research (Wash D C). 2023 Sep 15;6:0208. doi: 10.34133/research.0208 (PMC10503940; doi:10.34133/research.0208)
Supplement: Supplementary 1 — Fig. S1. The calculated Young’s modulus of Metglas as a function of applied magnetic field and stress field. Fig. S2. The stress distribution and the resonance response for traditional 2-1-typed and our proposed multilayered ME resonator. Fig. S3. Magnetic flux density distribution for the ME-MLTx. Fig. S4. The impedance and phase spectra for transmitter 2P-1M_10, 3P-2M_10 and 3P-2M_15. Fig. S5. Nonlinearity evolution. Fig. S6. Characterization of the nonlinearity and the radiation capability of the ME-MLTx. Fig. S7. Near-field radiation pattern in horizontal plane for ME-MLTx 3P-2M_15. Fig. S8. The overall transmitting and receiving scheme for BPSK modulation. Fig. S9. VLF communication implementation at 7-m distance by BPSK modulation with a symbol rate of 300 bps. Fig. S10. Influence of the data rate on obtained eye diagrams for BASK modulation. Fig. S11. Influence of the data rate on obtained eye diagrams for BPSK modulation. Fig. S12. The impedance curve for our used tuning coil. Fig. S13. Verification of long-distance VLF communication. Movie S1. Fifteen-meter VLF communication experiment. [file research.0208.f1.zip › Suporting Materials.docx]

Supplementary Materials

**Title**

**A multilayered magnetoelectric transmitter with suppressed nonlinearity for portable VLF communication**

**Authors**

Zhaoqiang Chu,^1,2,†^**^*^** Zhineng Mao,^1†^ Kaixin Song,^1†^ Shizhan Jiang,^1^ Shugang Min,^1^ Wei Dan,^1^ Chenyuan Yu,^1^ Meiyu Wu,^1^ Yinghui Ren,^1^ Zhichao Lu,^3^ Jie Jiao,^4^ Tianxiang Nan,^5^**^*^** Shuxiang Dong^6^**^*^**

**Affiliations**

^1^ Qingdao Innovation and Development Base, Harbin Engineering University, Qingdao 266071, China

^2^ College of Underwater Acoustics Engineering, Harbin Engineering University, Harbin 150001, China

^3^ Songshan Lake Materials Laboratory, Dongguan, Guangdong 523808 China

^4^ Shanghai Institute of Ceramics, Chinese Academy of Sciences, Shanghai 201800, China

^5^ School of Integrated Circuits and Beijing National Research Center for Information Science and Technology (BNRist), Tsinghua University, Beijing 100084, China

^6^ College of Engineering, Peking University, Beijing, 100871, China

**^*^**Correspondence should be addressed to : Zhaoqiang chu :zhaoqiangchu@pku.edu.cn; Tianxiang Nan: nantianxiang@mail.tsinghua.edu.cn Shuxiang Dong:sxdong@pku.edu.cn

^†^ These authors contributed equally: Zhaoqiang Chu, Zhineng Mao and Kaixin Song


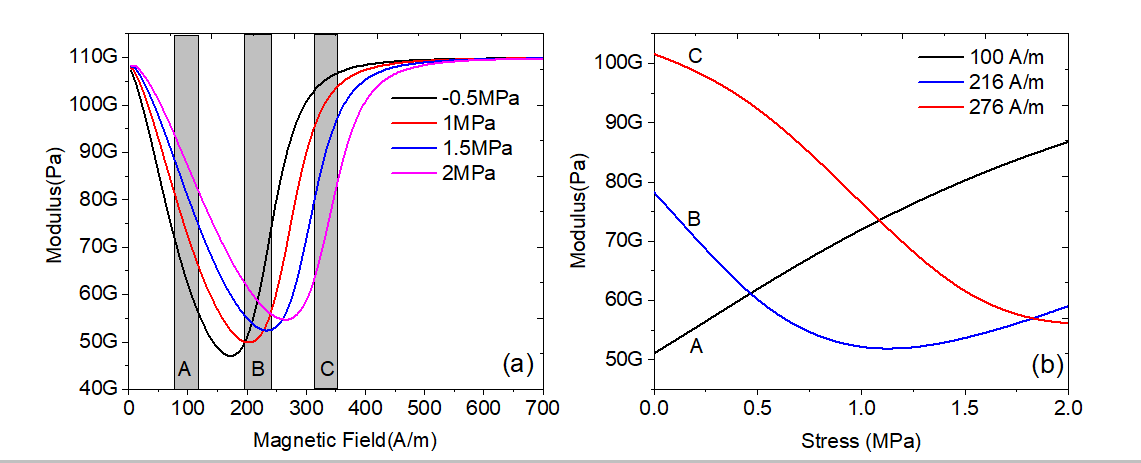


**Fig. S1 Delta-E effect in Metglas.** The calculated Young's modulus of Metglas as a function of applied magnetic field (a) and stress field (b). Here, we highlighted three regions. Region A performs a spring hardening effect and region C has a spring softening behavior. In region B, the Young's modulus just fluctuates within a narrow range. For a general ME devices, the optimized bias field normally lies in region A thus piezomagnetic laminates will contribute to the spring-hardening effect. s


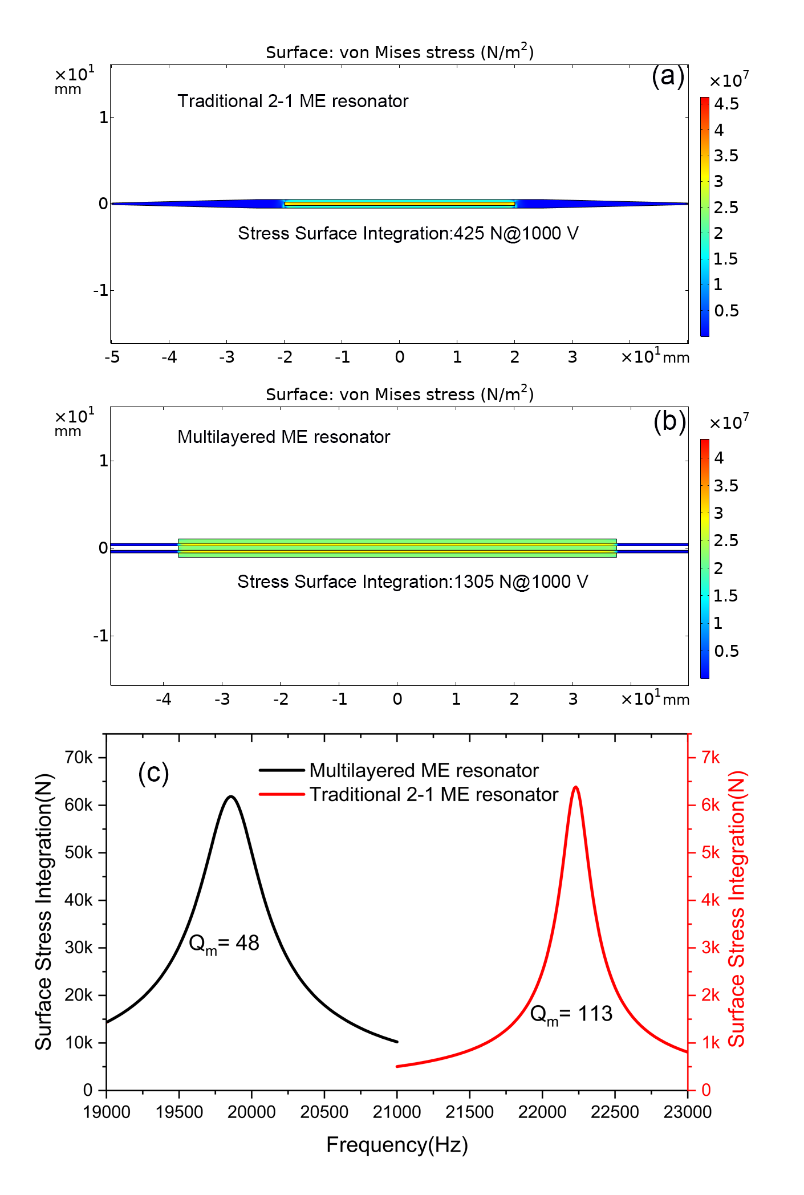


**Fig. S2. (a-b)** **The stress distribution for traditional 2-1 typed** (**a**) **and our proposed multilayered ME resonator** (**b**) simulated by stationary study. Here, the applied DC voltage is 1000 V. The derived surface integration of the stress in Metglas layers is 425 N and 1305 N for 2-1 typed and multilayered ME resonator, respectively. **(c)** The comparison of the surface stress integration in piezomagnetic layers between traditional 2-1 typed and our proposed multilayered ME resonator by frequency-domain study. Here, the mechanical quality factor for traditional 2-1 typed ME resonator and the multilayered ME resonator is set as 113 and 48, respectively, by considering the mechanical damping in COMSOL model.

It can be seen the transferred stress in the piezomagnetic layer of a multilayered ME resonator is obviously higher. With respect to the traditional 2-1 typed ME resonator, the piezoelectric core has a small dimension, which results in a poor driving force. In addition, bigger volume of piezomagnetic layer is permitted in a multilayered ME resonator and thus the working area can be enlarged dramatically. As a consequence, an improved stress transfer between two phases compared with a traditional 2-1 typed ME resonator is allowed.


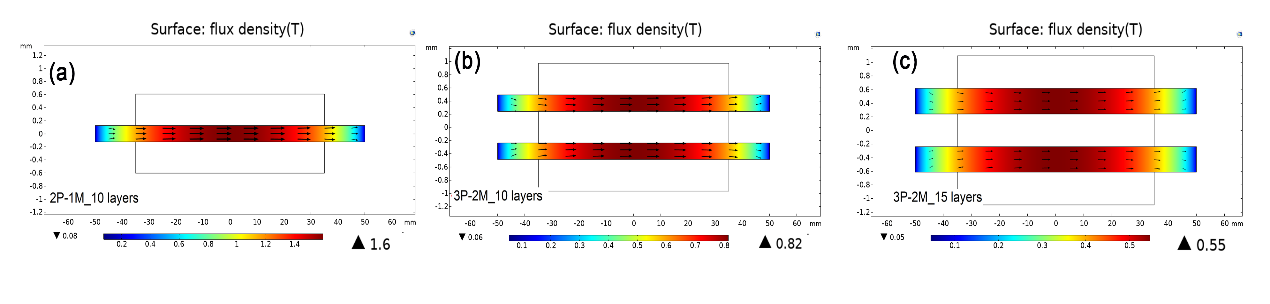


**Fig. S3. Magnetic flux density distribution for the ME-MLTx.** (**a-c**) Magnetic flux density in the longitudinal section for conventional sandwich-structured ME resonator 2P-1M_10 (**a**), a multilayer ME transmitter 3P-2M_10 with 10🞨 Metglas in each piezomagnetic laminate (**b**) and the proposed ME transmitter 3P-2M_15 with 15🞨 Metglas in each piezomagnetic laminate (**c**).


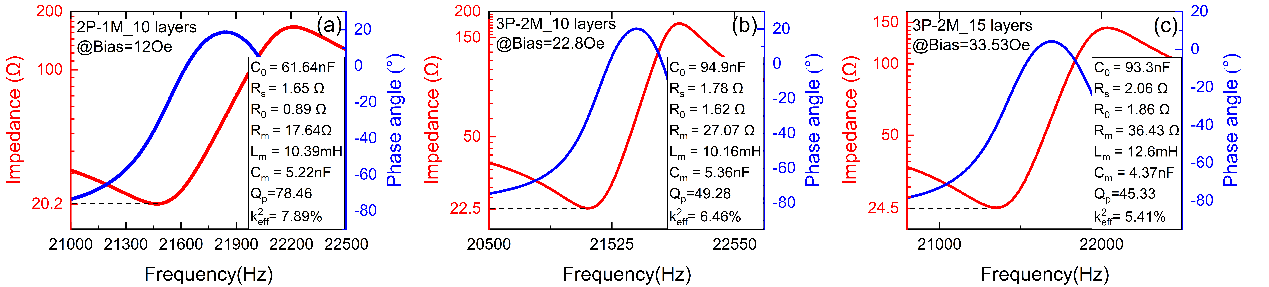


**Fig. S4.** (**a-c**) **The impedance and phase spectra for transmitter 2P-1M_10** (**a**), **3P-2M_10** (**b**) **and 3P-2M_15** (**c**). The measured value was obtained by 4294 impedance analyzer. The computed data was fitted from modified Butterworth–van Dyke (MBVD) mode. Based on the modified Butterworth–van Dyke (MBVD) mode, the calculated Q-value is 78.46, 49.28 and 45.33 for three kinds of transmitter. Normally, high-Q resonant is desired for efficient field radiation. But high-Q value also results in narrow bandwidth and strong nonlinearity. Hence, a balance should be considered and the suppressed nonlinearity needs to be satisfied first for generating strong magnetic field.


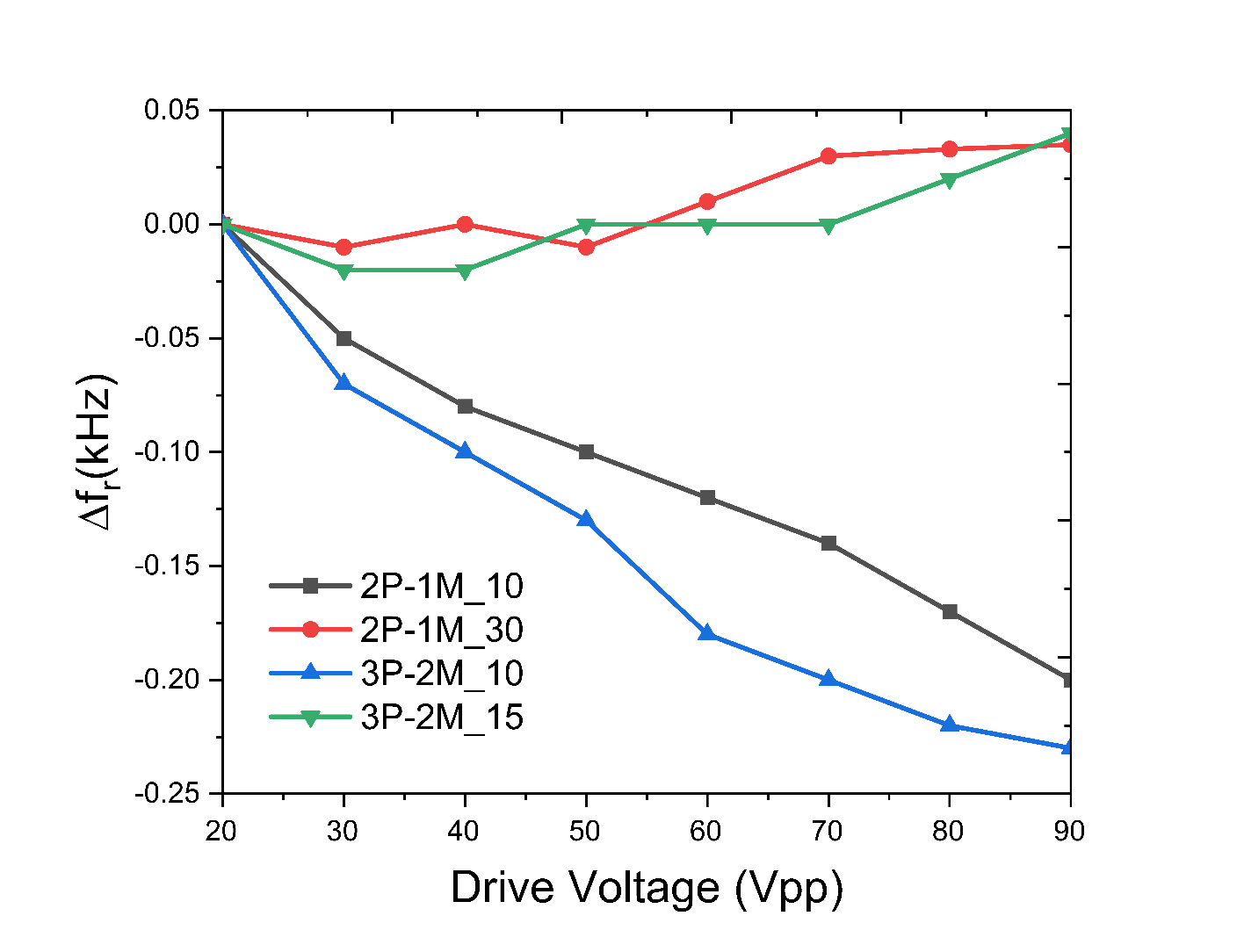
**Fig. S5.** **Nonlinearity verification.** The measured resonance frequency shift as a function of the amplitude of the burst signal for four kinds of transmitters (2P-1M_10, 2P-1M_30, 3P-2M_10, 3P-2M_15).


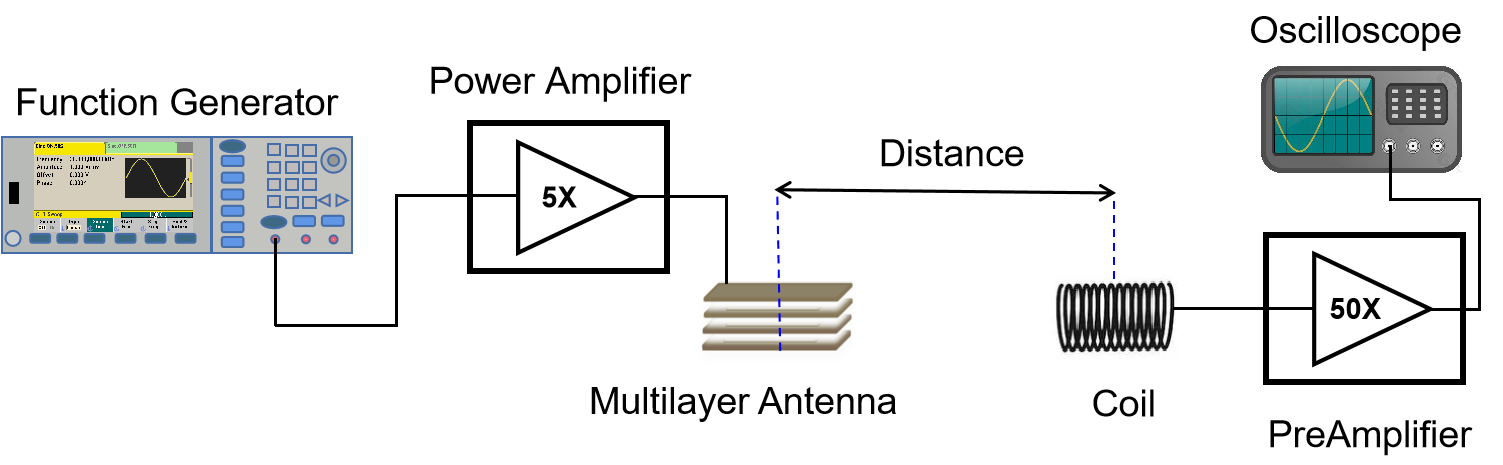


**Fig. S6. Characterization of the nonlinearity and the radiation capability of the ME-MLTx**

The graphical setup for measuring the radiation capability of a ME transmitter. Here, a waveform generator is used to first produce the targeted burst signal. The cycle number and the period of the burst signal is 50 and 500 ms, respectively. Then the burst signal is fed to a linear power amplifier before driving the ME-MLTx.. An air-core coil is used as the receiver. The induced voltage in the coil receiver is first passing through a voltage preamplifier and then recorded by an oscilloscope. The gain the passband is set as 50 and 3-100 kHz.

**
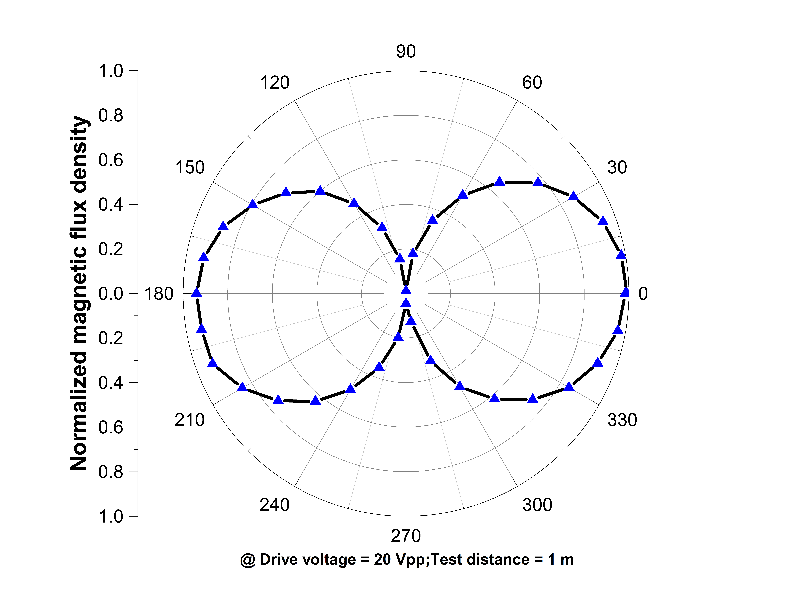
**

**Fig. S7 Nearfield radiation pattern in horizontal plane for ME-MLTx 3P-2M_15** under 20 Vpp excitation. Here, a perfect splay pattern is performed, which means a large angle coverage can be allowed.


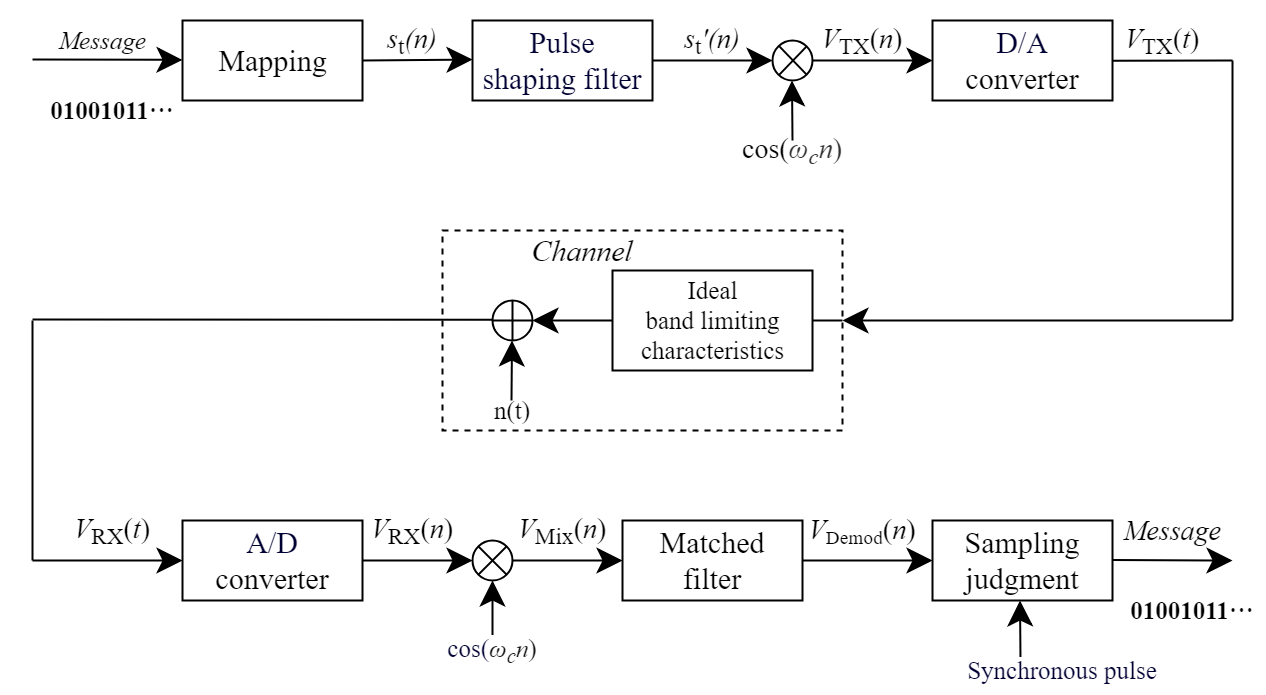


**Fig. S8.** **The overall transmitting and receiving scheme for BPSK modulation.**

At the transmitting side, the binary message is first transferred to single polarity non-return-to-zero (NRZ) pulse train *s*_t_(*n*) in the case of BASK modulation or to double polarity NRZ pulse train for BPSK modulation. Then a shaping filter is utilized to match the ring-down characteristic of the ME-MLTx as given in Figure 3f. In this case, the digital modulation signal *V*_TX_(*n*) can be generated by multiplying the digital carrier signal cos(*ω*_c_*n*) and digital baseband signal *s*_t_*'*(*n*). Finally, the analog modulation signal *V*_TX_(*t*) produced by the digital-to-analog converter is sent to the ME-MLTx after passing through the power amplifier. At the receiving side, a coherent demodulation is implemented to obtain the transmitted binary message. The signal received by the coil receiver is output to the oscilloscope after passing through a voltage preamplifier and the digital received signal *V*_RX_(*n*) is saved in the storage medium. The digital mixing signal *V*_Mix_(*n*) is generated by multiplying the digital carrier signal cos(*ω_c_n*) and the digital received signal *V*_RX_(*n*). And then a matched filter is performed on the digital mixing signal *V*_Mix_(*n*) before the sampling judgement to maximize the signal-to-noise ratio (SNR) and to reduce the intersymbol interference. Finally, the binary message is obtained by judging the digital demodulated signal *V*_Demod_(*n*) at the maximum value of the symbol by the synchronous pulses.


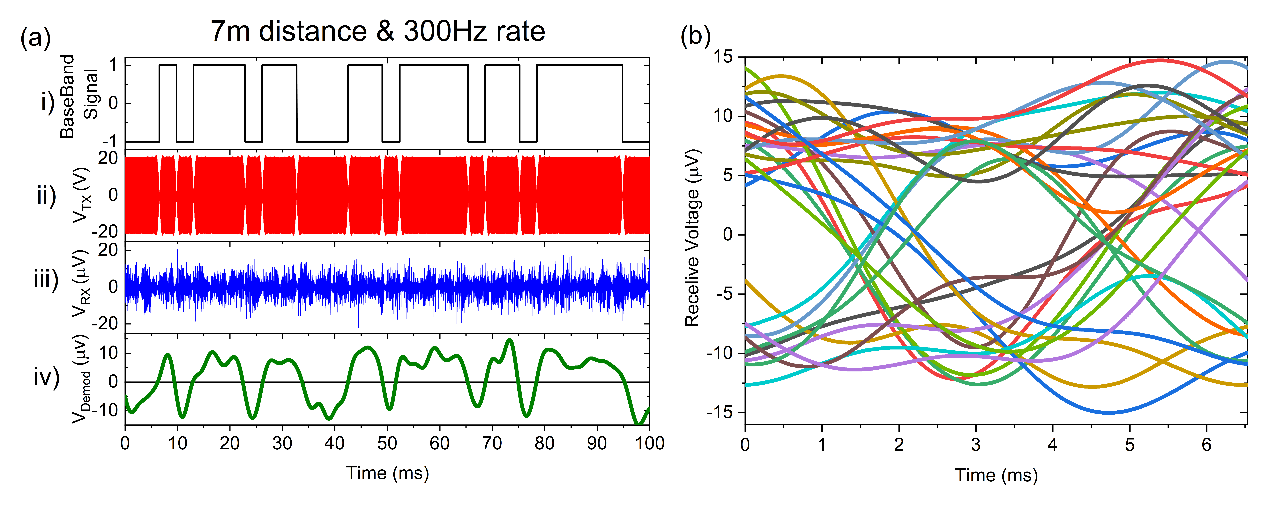


**Fig. S9.** **VLF communication implementation** **at 7 m distance by BPSK modulation with a 300 Hz symbol rate**. (a) The recorded i) binary baseband signal $s_{t}\left( n \right)$ with a symbol rate of 300 Hz, ii) the modulated transmitting signal $V_{TX}\left( n \right)$, iii) the received signal from the coil receiver $V_{RX}\left( n \right)$ and iv) the demodulated voltage signal $V_{Demod}\left( n \right)$. (b) The eye diagrams of the demodulated voltage signal $V_{Demod}\left( n \right)$.


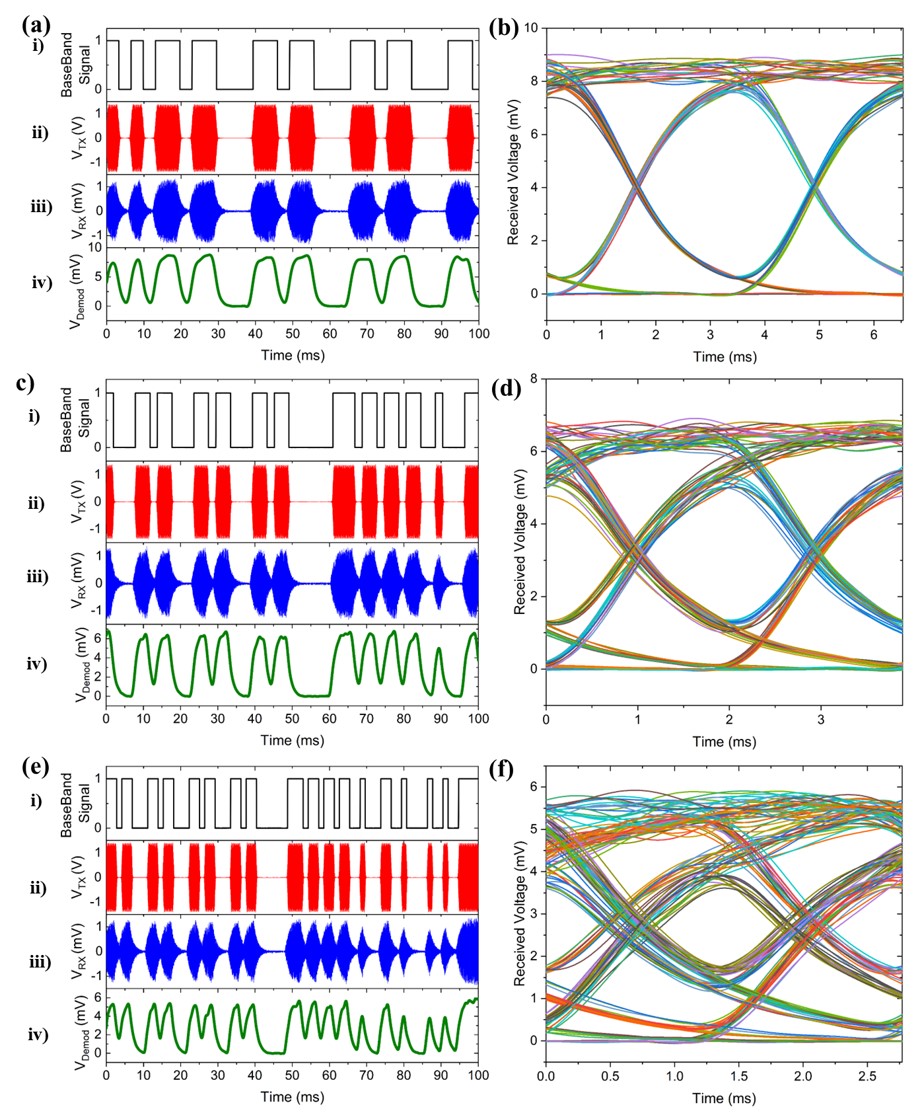


**Fig. S10. Influence of the data rate on obtained eye diagrams for BASK modulation..** VLF communication implementation at 0.3 m distance by BASK modulation. The recorded time-domain signal (**a,c,e**) and the corresponding eye diagram (**b,d,f**) for 300 Hz,500 Hz and 700 Hz data rate. Here, the ME-MLTx is kept operating in a linear condition with a weak driving voltage. When increasing the symbol rate from 300 Hz to 700 Hz, the SNR is dramatically decreased and large time variation of zero crossing is resulted both in BASK and BPSK mode as seen from the eye patterns in Supplementary Fig.10-11. Although the -3 dB bandwidth of the ME-MLTx reaches 500 Hz as shown in Figure 2c, such a high symbol rate is not supported because of the required excitation time and the ring-down time both exceeds 2 ms. Agreed with the comparison in Figure 6, however, BPSK scheme produces better communication performance compared with BASK scheme, especially in terms of the noise tolerance or the SNR.


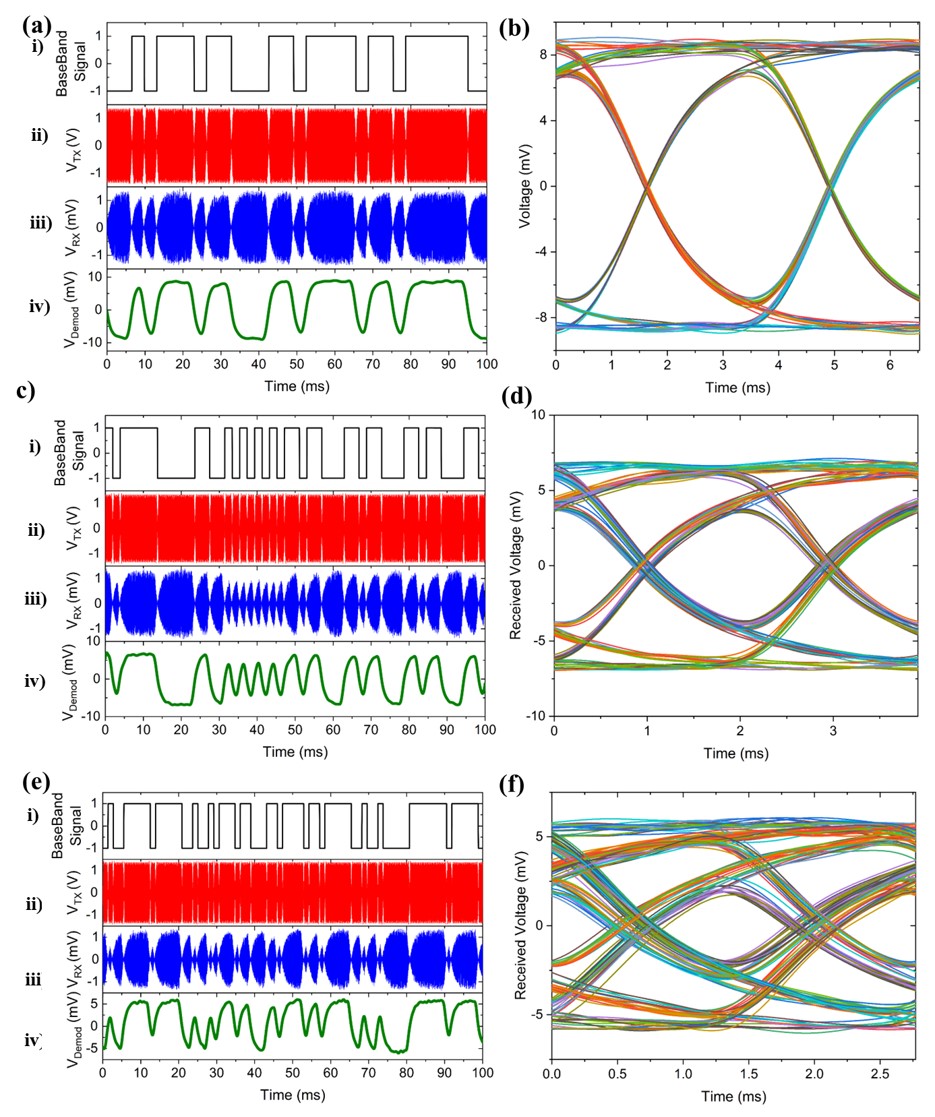


**Fig. S11. Influence of the data rate on obtained eye diagrams for BPSK modulation.** VLF communication implementation at 0.3 m distance by BPSK modulation. The recorded time-domain signal (**a,c,e**) and the corresponding eye diagram (**b,d,f**) for 300 Hz,500 Hz and 700 Hz data rate.


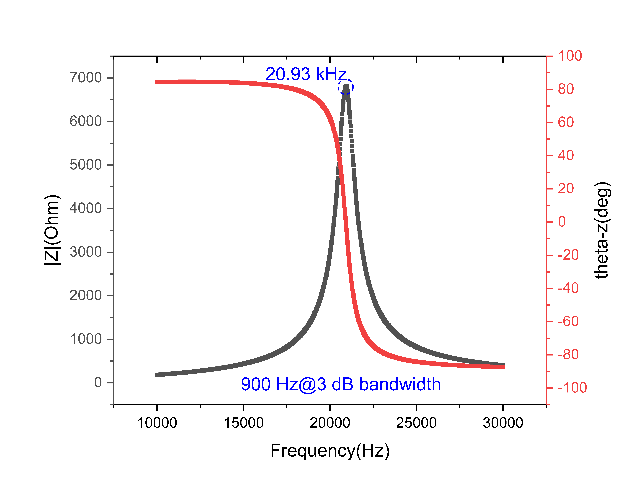


**Fig.S12 The impedance curve for our used tuning coil.**


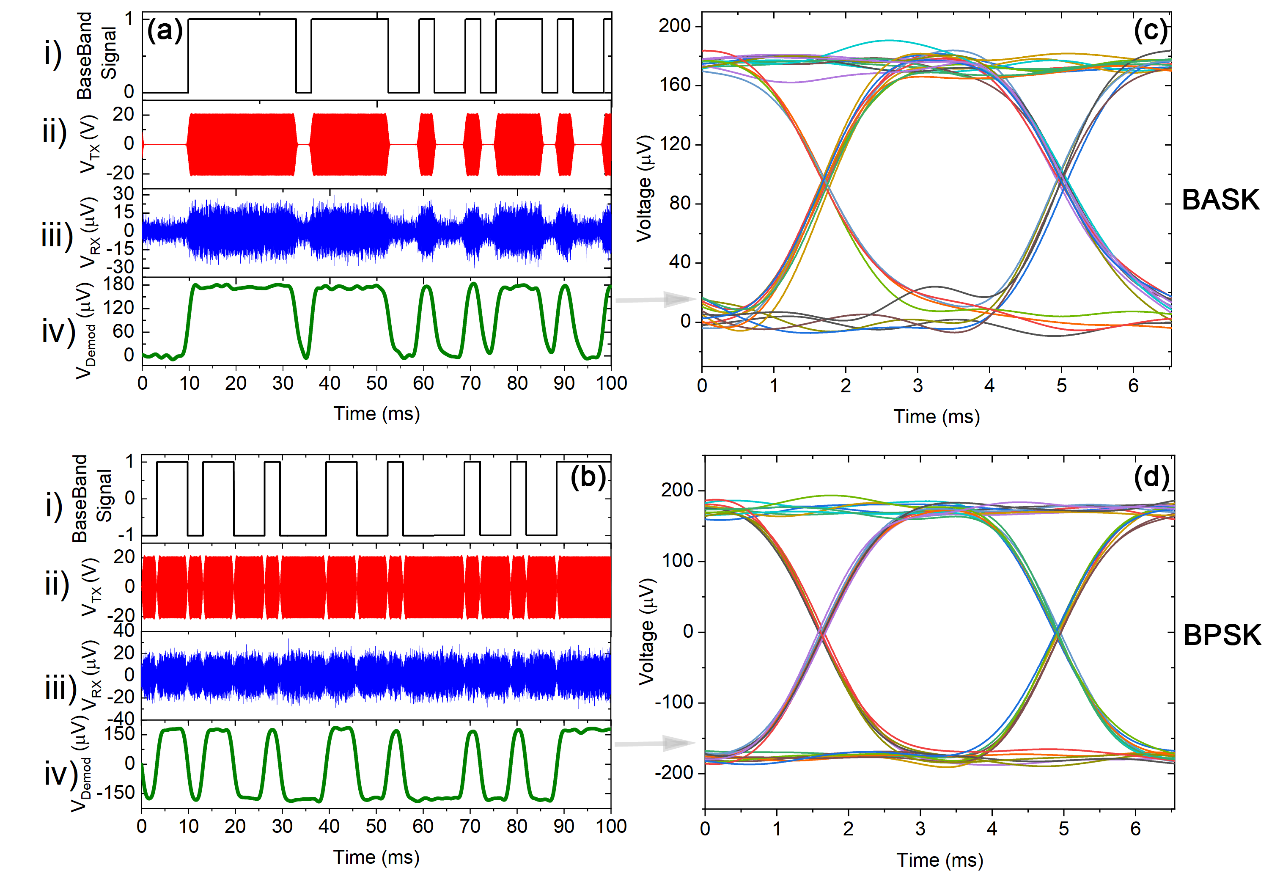


**Fig. S13. Verification of long-distance VLF communication.** VLF communication implementation at 15 m distance by BASK BPSK modulation with a 300 Hz symbol rate. **a,b,** The recorded i) binary baseband signal $s_{t}\left( n \right)$ with a symbol rate of 300 Hz, ii) the modulated transmitting signal $V_{TX}\left( n \right)$, iii) the received signal from the coil receiver $V_{RX}\left( n \right)$ and iv) the demodulated voltage signal $V_{Demod}\left( n \right)$ in the case of BASK modulation (**a**) and BASK modulation (**b**). **c,d,** The corresponding eye diagrams for the demodulated voltage signal $V_{RX}\left( n \right)$ via BASK modulation (**c**) and BASK modulation (**d**).

**Caption of Movies**

**Movie S1. 15-meter VLF Communication Experiment**

This movie first shows the building of the VLF communication system. Then, the real-time encoding and decoding results are given with the BASK and BPSK modulation, respectively.
